# The Diethylcarbamazine Delays and Decreases the NETosis of Polymorphonuclear Cells of Humans with DM Type 2

**DOI:** 10.1155/2020/4827641

**Published:** 2020-03-02

**Authors:** Juan C. Segoviano-Ramirez, Daniel F. Lopez-Altamirano, Jaime Garcia-Juarez, Juan E. S. Aguirre-Garza, Eloy Cárdenas-Estrada, Jesús Ancer-Rodriguez

**Affiliations:** ^1^Departamento de Patología, Facultad de Medicina, Universidad Autónoma de Nuevo León, UANL, Madero y Dr. Aguirre Pequeño, Mitras Centro, C.P. 64460, Mexico; ^2^Unidad de Bioimagen, Centro de Investigación y Desarrollo en Ciencias de la Salud, Universidad Autónoma de Nuevo León, UANL, Gonzalitos y Dr. Carlos Canseco, Mitras Centro, C.P. 64460, Mexico; ^3^Unidad de Ensayos Clínicos, Centro de Investigación y Desarrollo en Ciencias de la Salud, Universidad Autónoma de Nuevo León, UANL, Gonzalitos y Dr. Carlos Canseco, Mitras Centro, C.P. 64460, Mexico

## Abstract

Type 2 diabetes mellitus (DM2) is a disease that reports high morbidity and mortality rates worldwide. Between its complications, one of the most important is the development of plantar ulcers. The role of the polymorphonuclear cells (PMNs) is affected by metabolic diseases like DM2. Fifteen years ago, reports about a new mechanism of innate immune response where PMNs generate some kind of webs with their chromatin were published. This mechanism was called NETosis. Also, some researchers have demonstrated that NETosis is responsible for the delay of the ulcer healing both in patients with DM2 and in animal models of DM2. Purified PMNs from healthy and DM2 human volunteers were incubated with diethylcarbamazine (DEC) and then induced to NETosis using phorbol 12-myristate 13-acetate (PMA). In a randomized blind study model, the NETosis was documented by confocal microscopy. On microphotographs, the area of each extracellular neutrophil trap (NET) formed at different times after stimuli with PMA was bounded, and the intensity of fluorescence (IF) from the chromatin dyed with 4′,6-diamidino-2-phenylindole dihydrochloride (DAPI) was quantified. PMNs from healthy volunteers showed the development of NETs at expected times according to the literature. The same phenomenon was seen in cultures of PMNs from metabolically controlled DM2 volunteers. The use of DEC one hour before of the challenge with PMA delayed the NETosis in both groups. The semiquantitative morphometric analysis of the IF from DAPI, as a measure of PMN's capacity to forming NETs, is consistent with these results. The ANOVA test demonstrated that NETosis was lower and appeared later than expected time, both in PMNs from healthy (*p* ≤ 0.000001) and from DM2 (*p* ≤ 0.000477) volunteers. In conclusion, the DEC delays and decreases the NETosis by PMNs from healthy as well as DM2 people.

## 1. Introduction

Neutrophils or PMNs are the most abundant species of leucocytes in the human blood (60-70%) with a lifespan of 3 days. They have a granular cytoplasm whose diameter is between 9 and 12 micrometers (*μ*m) and a nucleus with thin recesses that confer it a lobulated aspect. The cytoplasmic granules are classified in primary or azurophilic ones (diameter = 0.5 *μ*m), containing enzymes with antimicrobial activity like the acid hydrolase, myeloperoxidase, lysozyme, cathepsin G, neutrophil elastase, and collagenase; secondary or specific ones (diameter = 0.1 *μ*m), containing antimicrobial molecules and some molecules that contribute with the migration of neutrophils; and tertiary ones whose main content is composed of gelatinase, cathepsin, and other glycoproteins that facilitate the phagocytosis. Their cellular membrane expresses adhesion molecules, like selectins and integrins, cytokine receptors, and receptors for the innate immune response: Toll-like receptors, receptors linked to protein G that participate in the host defense and activate the chemotactic migration and receptors for the Fc portion of the immunoglobulins, useful for the phagocytosis of immune complexes [1].

The role of the neutrophils is very important in the acute inflammation due to bacterial invasion, in which a wide variety of cells participates secreting molecules or expressing them on their membranes provoking the migration of PMNs to the site of infection where they recognize opsonized antigens by the immunoglobulins and phagocytes and destroy them by the lysosomal phagocytic pathway or by the respiratory burst. Once their job is done, the neutrophils suffer apoptosis or necrosis, and together with the bacterial debris, they form the pus which is eliminated by the macrophages [2–4].

In 2004, Brinkmann et al. described the formation of some fibrous extracellular structures after the liberation of chromatin and granules containing enzymes by the PMNs. He called them neutrophil extracellular traps (NETs) [5]. Since then, the process that leads to the formation of NETs is called NETosis. The NETs are made of molecules of deoxyribonucleic acid, histones, enzyme-like neutrophil elastase, and intact granules. Once the chromatin fibers are expulsed to the extracellular space, they trap the nearby bacteria immobilizing them and degrading their virulence factors. In this way, NETosis is considered as a new mechanism of the innate immune response.

From the ultrastructural point of view, NETs are fragile and smooth filaments of 17 nanometers (nm) in diameter containing globular domains of 25 to 50 nm in diameter. When they are formed into an aqueous medium, they hydrate themselves and drastically change their morphology into filaments forming clouds, reaching a size up to 10 times more than the neutrophils that originate them. In the presence of bacteria invading the connective tissue, neutrophils activate the classical pathway of defense to phagocyte and destroy them. Also, PMNs trigger a mechanism which is characterized by the disappearance of the nuclear recesses and disorganization of the nuclear envelope, followed by the fusion of both membranes and expulsion of chromatin to the cytoplasm. The homogeneous mixture of cytoplasmic and nuclear content increases the intracellular pressure compromising the integrity of the cellular membrane and induces the formation of a pore through which the said mixture is expelled to the extracellular space forming thin NETs that immobilize the nearby pathogens [6, 7].

The diethylcarbamazine (DEC) is a water-soluble derivate of piperazine, which is white, insipid, odorless, and stable to heat, and which is used as an antiparasitic drug. A dose-dependent immunomodulatory effect of the DEC on the phagocytic cells in an animal model was reported. Researchers found that low doses of DEC improve the cytokine production, while high doses improve the respiratory burst in neutrophils [8]. The same research group found that DEC improves the cellular innate immune response, since its administration achieved the regression of an inflammatory granulomatous process, experimentally induced with intracellular bacteria in animal models [9]. In a pilot study, recently, we demonstrated that DEC decreases the NET formation by PMNs of human volunteers challenged with PMA into *in vitro* assays [10].

Diabetes mellitus is a disease with a high rate of morbimortality in Mexico and worldwide. The American Diabetes Association establishes that, “The diabetes mellitus (DM) is a group of common metabolic disorders that share the phenotype of hyperglycemia.” There are various types of DM as a result of a complex interaction between genetics and environmental factors [11].

In the patients with long-time evolution of DM, the hyperglycemia chronic state induces the glycosylation of some serum proteins [12] and collagen fibers of the microvasculature [13]. This together with the decrease in phagocytic capacity constitutes a determinant point for the development of the chronic complications like nephropathy, cardiomyopathy, retinopathy, peripheral neuropathy, peripheral arterial disease, and diabetic foot [13]. In particular, the diabetic foot has a bad impact on the life quality of a lot of patients. In Mexico, it is present in 15 to 25% of the cases with DM [14], and recently, it is considered one of the first causes of nontraumatic amputation [15]. This complication is originated in some point of pressure in the soles, where tissue hypoxia and ischemia due to microangiopathy cause formation of ulcers. Due to the bad scarring, these ulcers may progress to osteomyelitis, and the probability of amputation of the involved extremity goes up, which leads to disability and early death [16].

Groups of researchers have documented the presence of NETs in wounds, both in diabetic humans and murine models of DM types 1 and 2. They attribute the delay in the scarring of wounds, in diabetic people, to the NET formation because their neutrophils are more susceptible to NETosis for presenting an increase of expression of peptidyl arginine deiminase 4 (PAD), an enzyme that participates in the chromatin decondensation. These researchers find a delay in scarring of experimental wounds of diabetic wild type mice as well as large quantities of NETs and high levels of citrullinated histone H3, which was not observed in Padi4-/- mice. They showed that the use of DNAase cuts off the NETosis speeding up wound healing and improving the NET-dependent scarring, both in diabetic and in wild type normoglycemic mice. For the above, they proposed that inhibition of the NETosis in DM2 improved the scaring of the wounds due to the reduction of the chronic inflammation induced by NETs [17]. In this study, we sought to demonstrate the immunomodulatory effect of DEC on the NETosis in PMNs from humans with DM2 *in vitro* assays. The enlightenment of this phenomenon might have clinic applications in the control of wounds and plantar ulcers in patients with DM2 and offer them benefits both in the costs of its treatment and in their life quality.

## 2. Materials and Methods

### 2.1. Sample Size

With previous informed consent, 7 healthy adults and another 7 adults with DM2 diagnosis, medical treatment, and metabolic control were recruited [18]. For all of them, a general clinical history was made, and for those with DM2 diagnosis, a determination of glycosylated haemoglobin was done. In both groups of volunteers, only those without smoking and alcoholism background, any inflammatory or infectious active processes, or who had not taken any nonsteroid anti-inflammatory or anticoagulant drug were included. Those volunteers who at the moment of the recruitment had oedema in any part of the body or had any clinical data of anaemia, dehydration, diagnosis of heart disease, arterial hypertension, heart failure, asthma, epilepsy, pregnant, or in lactation were also excluded. The volunteers who prior to blood extraction requested their voluntary withdrawal of the study were eliminated, as well as those who had an incomplete file or did not follow the instructions given during the recruitment. Finally, those volunteers whose blood samples were contaminated during the purification process or whose total count of purified PMNs was outside of the normal limits were eliminated.

### 2.2. PMN Purification

The PMNs were purified from peripheral venous blood in EDTA, according to the standardized protocol previously described by Brinkmann et al. [19]. Briefly, 24 ml of human blood with EDTA was collected and distributed in four Falcon tubes, then 6 ml of Histopaque®-1119 (Sigma-Aldrich, cat no 11191) was added to each tube, and the sample was centrifuged at 800 × g for 20 minutes. Yellowish and clear top layer from each tube was aspirated and discarded. Lower reddish phases containing granulocytes were transferred into new Falcon tubes. 8 ml of 1 M PBS (pH 7.4) was added and then centrifuged for 10 mins at 300 × g to wash the cells. Meanwhile, two Falcon tubes were prepared with a gradient filling them with solutions of Percoll (GE Healthcare, cat no 17-0891-02) at 85%, 80%, 75%, 70%, and 65%, layering 2 ml of every percentage on top of each other in decreasing order. After the centrifugation, pellets were combined and the sediment cells were resuspended with 4 ml of PBS, then 2 ml of the resuspension was layered into each of the gradient tubes and centrifuged for 20 mins at 800 × g. After the centrifugation, the top layer and most of the 65% layer were removed; the white remaining interphases until the 85% layer were collected into new Falcon tubes. The Falcon tubes were filled with PBS and centrifuged for 10 minutes at 300 × g to wash the cells. Then, the supernatant was removed, and the sedimented cells (usually >95% are PMNs) were resuspended in 2 ml of PBS. After that, the cells were counted by a portable automated cellular counter (Scepter TM, Millipore).

### 2.3. PMN Incubation

In 24-well plates (Corning, cat no 3524), six wells were grouped into one of three groups: positive control, negative control, and experimental. Into each well, containing 500 *μ*l of RPMI 1640 (Life Technologies/Thermo Fisher, cat no 11875085) supplemented with 2% (final concentration) of human serum albumin (Sigma-Aldrich, cat no A6784), a sterile circle borosilicate cover slip (Thermo Fisher®, 15CIR-1) was introduced and 2X105 PMNs from healthy volunteers were seeded. Simultaneously, the same incubation with the PMNs from the volunteers with DM2 was made. In the wells of the experimental group, 100 *μ*M (final concentration) of DEC was added (Sigma-Aldrich, cat no D8765) and cells were incubated for 1 hour. After the incubation time, 600 nM (final concentration) of PMA (Sigma-Aldrich, cat no P1585) was added to the experimental and the positive control groups to induce the NETosis. NET formation was stopped via chemical fixation with 4% paraformaldehyde (Sigma-Aldrich, cat no P6148) in 1 mM, pH 7.4 PBS, at 0, 15th, 30th, 60th, 120th, and 180th minutes.

### 2.4. Immunostaining

Over coverslips containing the incubated cells, the indirect immunofluorescence staining technique was done. Rabbit anti-neutrophil elastase primary antibodies (Abcam, ab68672) and donkey anti-rabbit IgG conjugated with Alexa Fluor® 488 (Abcam, ab150073) secondary antibodies were used. The chromatin was stained with DAPI included in an antifade mounting media (VECTASHIELD HardSet, Vector Laboratories H1500). Coverslips were mounted over standard borosilicate slides (Corning, 2947BC).

### 2.5. Documentation by Confocal Microscopy

The morphological studies were realized with fluorescence microscopy and multiphoton confocal microscopy. The image acquisition for morphometric studies was realized with a confocal scanner (Zeiss LSM710 NLO), through the ZEN2009® software following a blind randomized systematic method of sampling; five microphotographs per time by each group were taken. Microphotographs were taken by a person who did not know which group was being analyzed and which treatments were given to each culture. Positioning of the microscope's objective over the slide was done without observing through the oculars, moving randomly the stage and acquiring immediately the microphotograph just at the point where it was stopped.

Into each image, using the DAPI's channel, the PMNs with morphological data of activation and progress towards NETosis or the presence of NETs according to Brinkmann and Zychlinsky's criteria was identified [20]. The onset time of NET formation in each group was recorded. This last was identified by the presence of a chromatin net joined to the neutrophil elastase, both evidenced by colocalization of their fluorochromes: DAPI and Alexa Fluor 488, respectively.

Delimiting the profiles of the activated PMNs and those PMNs who were exhibiting the formation of the NETs in each microphotograph, the quantitation of DAPI's intensity of fluorescence (IF) was done. The value of DAPI's IF per incubation time, group, and population type was recorded in a database.

### 2.6. Statistical Analysis

The normal distribution of the IF of the DAPI using the Kolmogorov-Smirnoff test as well as the Shapiro-Wilk test with fixed significance was analyzed. To determine the effect of the DEC over the NETosis and establish statistic differences between experimental groups into healthy and DM2 populations as well as between them, the IFs from DAPI for each time and group were compared with a repeated measure analysis of variance, fulfilling all and every one of the additional requirements of the tests. The NCSS® package was used for all statistical analysis.

## 3. Results

The evidence of the DEC's effect over NETosis *in vitro* was obtained by the morphological analysis of PMNs and NETs found in the confocal microphotographs and by the semiquantitative morphometric analysis of the DAPI's IF of such profiles, once they were delimited over the microphotographs.

### 3.1. Morphological Analysis

**Figure 1:**
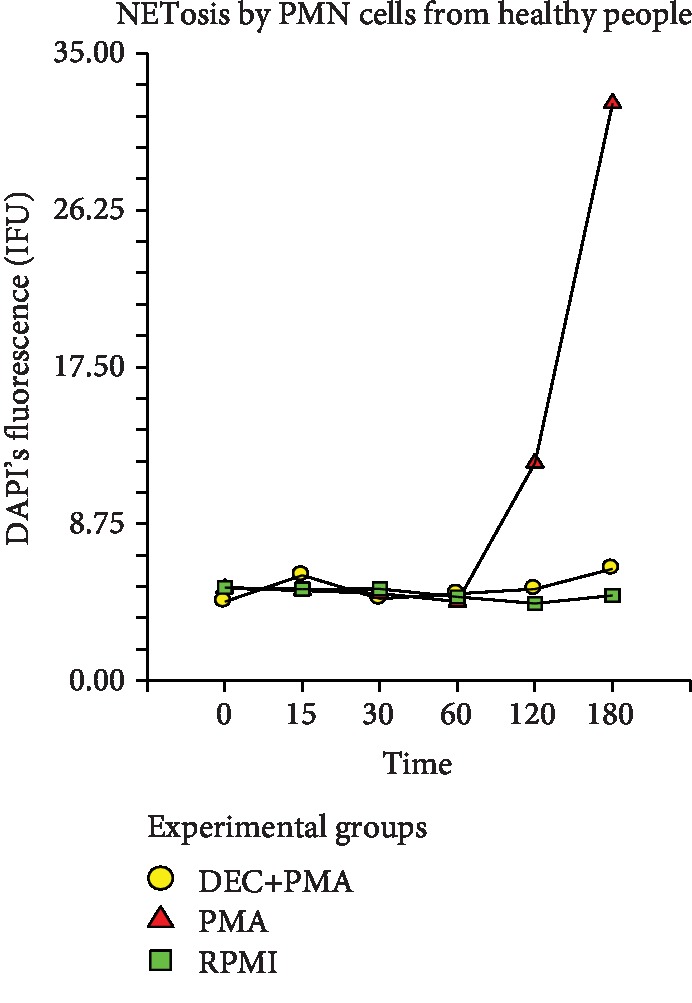
NETosis shown by PMNs from healthy volunteers indirectly measured by DAPI's intensity of fluorescence (IF U). PMA: control positive group; DEC+PMA: experimental group; RPMI: negative control group. Repeated measure analysis of variance. The marks represent the average of 5 measurements of IF in seven separate sample runs (*n* = 35).

**Figure 2:**
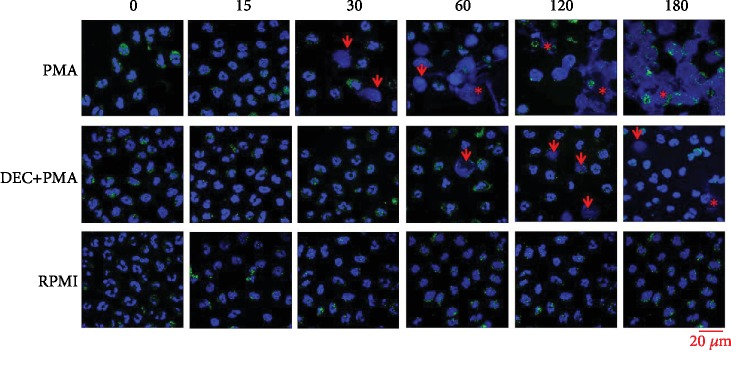
Morphological analysis of NETosis shown by PMNs from the healthy volunteer group. Photocomposition of confocal microphotographs of PMNs stained with indirect immunofluorescence technique with rabbit monoclonal anti-NE antibodies and mouse polyclonal anti-rabbit IgG antibodies conjugated with Alexa Fluor 488 (green color) and DAPI (blue color). PMA (positive control), DEC+PMA (experimental group), and RPMI (negative control) at 0, 30, 60, 120, and 180 minutes post challenge with PMA. In the positive control group, functional profiles of PMNs are present from 0 till the 15^th^ minute, some activated PMNs with rounded aspect (arrows) appear at the 30^th^ minute, and numerous NETs (asterisks) with reticular appearance can be seen from the 60^th^ minute to the end. In the experimental group, functional profiles of PMNs are present from 0 till the 30^th^ minute, and just some PMNs with morphological data of activation (arrows) are present from the 60^th^ till the 180^th^ minute. In the negative control group, all profiles of PMNs have a morphological aspect of functional cells along the studied times. Multiphoton confocal microscopy, 63x; lasers: 405 and 488 nm.

Since the time 0 until the 15th minute poststimulation, PMNs from healthy volunteers that were incubated with PMA (positive control) exhibited an aspect which corresponds to normal functional cells: rounded profiles with a multilobulated nucleus and two or more recesses. Towards the 30th minute, some cells showed morphological characteristics of activation, like nuclei in a band, due to the loss of its recesses or with a round aspect occupying the most of the cytoplasm and the apparent lowering of the azurophilic granules; this last aspect was caused by the disorganization of the nuclear envelope and the fusion of their nuclear membranes (Figure 1). It should be noted that at this moment, only some PMNs showed the formation of NETs. Instead, it was up to the 60th minute when the largest number of NETosis was observed, increasing until the 120th minute. At the 180th minute poststimulation with PMA, the formation of NETs generally appeared in all the rest of the PMNs found in the microphotographs. Otherwise, the PMNs incubated with DEC and later challenged with PMA (experimental group) showed the characteristic morphology of functional cells, from time 0 until the 30th minute in all the analyzed microphotographs and only in some of those scarce activated PMNs were appreciated showing morphological characteristics of activation toward NETs formation. By the 60th minute poststimulation, some of the PMNs started to exhibit morphological data of activation characterized mainly by nuclear disorganization. This same phenomenon was observed in PMNs in the 120th and 180th minutes. Although the number of profiles with nuclear disorganization was slightly rising between the 60th and 180th minutes, the presence of NETs was sporadic. On the contrary, the PMNs found in the group incubated with RPMI (negative control) maintained the classical morphological architecture of a functional cell at all times (Figure 2).

**Figure 3:**
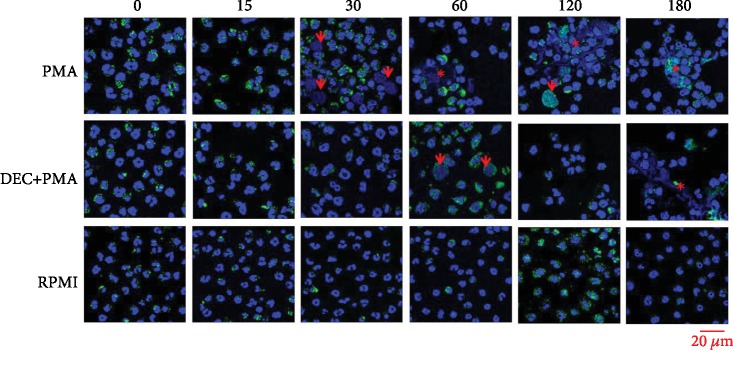
Morphological analysis of NETosis shown by PMNs from the diabetes mellitus volunteer group. Photocomposition of confocal microphotographs of PMNs stained with indirect immunofluorescence technique with rabbit monoclonal anti NE antibodies and mouse polyclonal anti-rabbit IgG antibodies conjugated with Alexa Fluor 488 (green color) and DAPI (blue color). PMA (positive control), DEC+PMA (experimental group), and RPMI (negative control) at 0, 30, 60, 120, and 180 minutes post challenge with PMA. In the positive control group, the PMNs show the morphological aspect of activation (arrows) at the 30^th^ minute and NETosis (asterisks) is observed from the 60^th^ minute till the end. In the experimental group, PMNs show morphological data of activation at the 60^th^ minute and NETs can be observed till the end of the experiment. Finally, in the negative control group, the cells show morphological characteristics of functionality in all studied times. Multiphoton confocal microscopy, 63x; lasers: 405 and 488 nm.

The morphological analysis of purified PMNs from DM2 volunteers showed, in the positive control group, the aspect of normal functional cells from the minute 0 till the 15th minute poststimulation: multilobed nucleus, keeping their recesses. At the 30th minute, they exhibited some morphological data of activation characterized by loss of recesses and a change of the nuclear profile from multilobed to circular. Additionally, at the 60th minute, they presented the low presence of filamentary, reticular, extracellular, and DAPI-positive structures, associated with AF488-positive granules (it means NETs). At the 120th and 180th minutes, the presence of NETs in more abundant aggregates accompanied by some activated PMNs is noticeable. Otherwise, the experimental group started to present activated neutrophils until the 60th minute poststimulation, and by the 120th minute, the NET formation was present and discreetly rose up to the 180th minute. It should be mentioned that in these last two times, the presence of NETs was very scarced in comparison with the positive control group of healthy volunteers. Finally, the PMNs of the negative control group presented morphological characteristics of normal functional cells in all analyzed times (Figure 3).

### 3.2. Morphometric Analysis

The tendency towards NETosis, measured indirectly through the IF from DAPI staining the chromatin of the NETs, confirmed the morphological findings in our experiments, both in the group of healthy volunteers and in the volunteers with DM2. In the healthy volunteers, the statistical analysis of such IF, per times and by treatment groups, did not show a significant difference between the positive control, the negative control, and the experimental groups since the time 0 up to the 60th minute poststimulation with PMA (*p* ≤ 0.5873) (Figure 1). The IF in the positive control group increased abruptly between the 60th and 120th minutes and continued with a highly marked slope until the 180th minute. Instead, in the experimental and negative control groups, the registered IF maintained dejected for all the analyzed times, without finding a difference that was statistically significant between them. The statistic comparison between the three groups of treatment including all times (0 till 180th minute) demonstrated that a statistically significant difference exists between them. (ANOVA, *p* ≤ 0.00001) (Figure 1).

**Figure 4:**
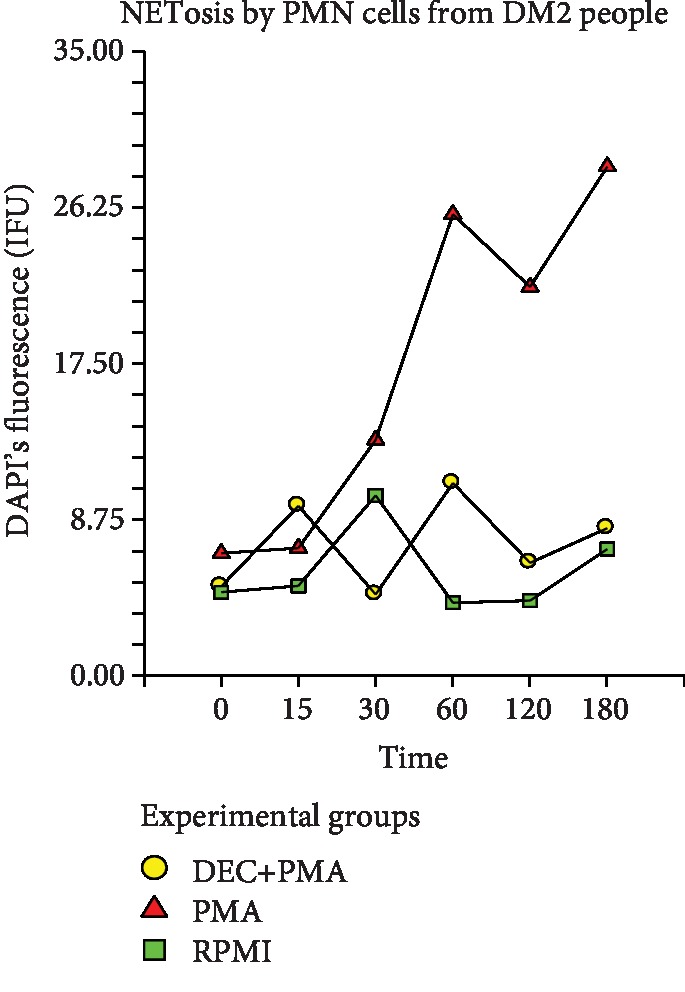
NETosis shown by PMNs from DM2 volunteers indirectly measured by DAPI's intensity of fluorescence (IF U). PMA: control positive group; DEC+PMA: experimental group; RPMI: negative control group. Repeated measure analysis of variance. The marks represent the average of 5 measurements of IF in seven separate sample runs (*n* = 35).

**Figure 5:**
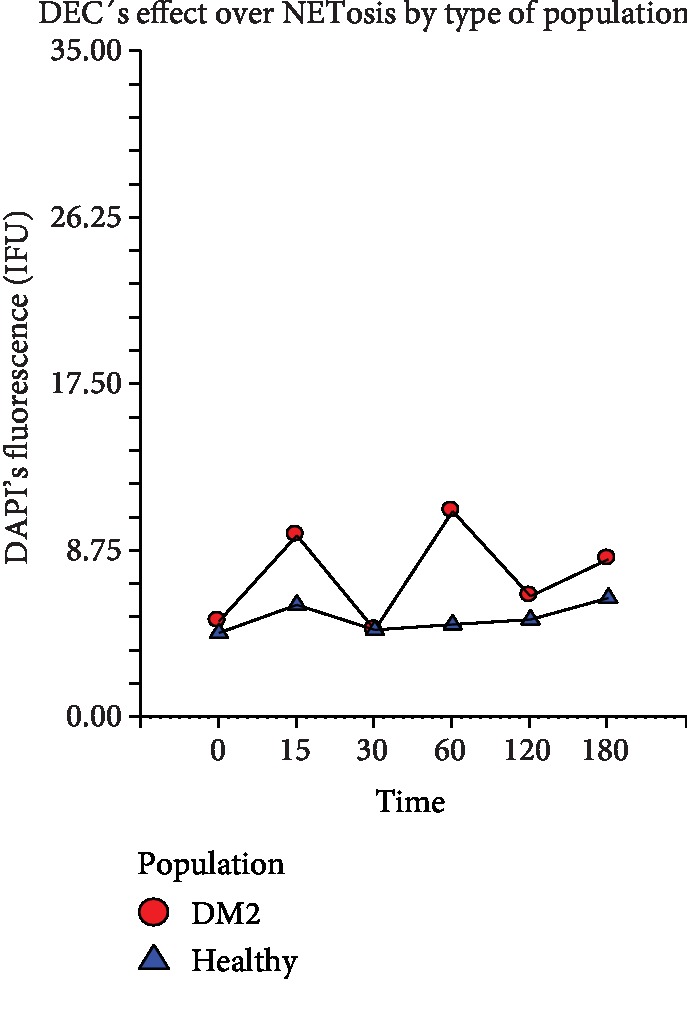
NETosis shown by PMNs after incubation with DEC at 0, 15, 30, 60, 120, and 180 minutes post challenging with PMA and indirectly measured by DAPI's intensity of fluorescence (IF U). DM2: diabetic volunteer group; healthy: healthy volunteer group. Repeated measure analysis of variance. The marks represent the average of 5 measurements of IF in seven separate sample runs (*n* = 35).

In the same way, the morphometric analysis realized to the PMNs purified from the DM2 volunteers showed that the intensity of the fluorescence registered did not present any statistically significant difference since the time 0 up to the 30th minute poststimulation in all the treatment groups (*p* ≤ 0.0582) (Figure 4). Instead, such IF, as an indirect indicator of NETosis, again showed an abrupt upward trend in the group treated with PMA (positive control) at the 60th minute post activation and kept increasing progressively until the 180th minute. In another way in the experimental group (DEC+PMA) and the negative control (RPMI), the intensity of fluorescence stayed low in all analyzed times without finding a significant difference between them. Comparing the IF of the three groups of treatment through all the analyzed times, again, the statistical analysis showed that there exists a significant difference between the negative control and the experimental group versus the positive control group (*p* ≤ 0.0004) (Figure 4). Furthermore, when comparing the effect of DEC on the NETosis exhibited by the PMN cells from volunteers with DM2 against that of healthy volunteers, we did not find statistical differences between both cell populations (Figure 5).

## 4. Discussion

In 2004, Brinkmann and collaborators reported for the first time the NET formation in PMNs as an extracellular defense mechanism that makes part of the innate response against components of the bacterial cell wall like LPS, mannose, and PAMPs, as well as fungi and parasites [5]. Later evidence showed that this mechanism is also performed by other cell types [20].

Although the best *in vitro* inductor for the NET formation is the PMA, a synthetic analog of LPS, nowadays, numerous *in vitro* NET inductors of endogen origin are known, such as proinflammatory cytokines (IL-6, IL-8, and TNF-*α*), glucose, activated platelets, and hydrogen peroxide, even though there is evidence indicating that some of them lack activity in vivo [21]. Nevertheless, the NETosis can become deleterious to the host himself since it has cytotoxic characteristics.

In the present study, we found morphological evidence of activation in some PMNs from healthy volunteers after being 30 minutes post challenged with PMA (positive control group). In the 60th minute, the activation of the rest of the PMN and the onset of NETosis were evidenced; this activation increased exponentially at the 120th and reached its maximum value at the 180th minute. These findings match with those described by Brinkmann and Zychlinsky [20] who showed the morphological changes in the activation inside the first 60 minutes. Several studies have reported that the time it takes for a neutrophil to form NETs varies depending on the type and concentration of the inducer. One of them reported that the period of induction of NETosis using 100 *μ*M PMA varies from 10 minutes up to 24 hours [22–24]. In other studies, in which PMA was used at concentrations between 120 and 1620 nM, the NETs were present between 10 minutes and 4 hours postactivation. Our study included a dose that fits between these ranks, so it is possible that the few NETs observed at the 30th minute were due to the used concentration of PMA, as observed by Munafo et al. [25] and Skopelja et al. [26]. It should be noted that they obtained this data from studies *in vitro* in which they identified as NETs those profiles of PMNs presenting the initial morphological changes: loss of the recesses, spheroidal structure of the nucleus, and the reticular structure of the NET itself. In contrast, we identified as NETs the reticular structure of DAPI-positive chromatin, accompanied by NE granules stained with AF488 and recorded the moment in which those structures were visualized. Also, it is possible that the NETs we found at the 30th minute were formed by those PMNs which were previously stimulated by another inductor inside the bloodstream before its extraction. New experiments that contemplate the purification of naïve PMNs will be necessary. These will allow us to discriminate and purify the functional PMNs from those previously activated into the bloodstream and in this way ensure that NETosis is a consequence of the experimental inductor and not of some spurious stimuli.

On the other hand, although PMNs from healthy volunteers that were incubated with 100 *μ*M DEC before stimulation with PMA (experimental group) showed morphological aspects of activation between the 60th and 120th minutes and NETosis till the 180th minute, this latter was present at a lower amount in relation with the positive control group. Considering the immunomodulatory effect that DEC has over the cytokine production and the respiratory burst in the PMN [8] as well as over the proliferation of lymphocytes [9], one would expect that the DEC had some modulatory effect over the NETosis. We know that DEC by itself does not induce NETosis, since in our control experiments, when we incubated the PMNs with DEC while lacking PMA, we could not observe it. We found similar results in a previous pilot study implemented with PMNs from healthy young volunteers [10].

In the case of PMNs from diabetic volunteers, those challenged with PMA (positive control group) showed similar behaviour like that showed by the PMNs from healthy volunteers. The morphological changes corresponding to the activation defined by Brinkmann and Zychlinsky [20] were present at the 30th minute. The phenomenon of the NETosis started to present itself from the 60th minute onwards, increasing as time progresses (Figure 3). This might mean that, although in the diabetic people there exists a state of phagocytic immunodeficiency, when they are in the metabolic control, their PMNs will exhibit NETosis in a very similar way to that one showed by the PMNs of healthy subjects. In the future, studies searching the correlation between glycosylated haemoglobin values and the tendency toward NETosis by the PMNs from patients with DM2 are needed.

On the other hand, those PMNs of diabetic volunteers that were incubated with DEC and later challenged with PMA (experimental group) showed morphological characteristics of activation up to the 60th minute and the NETs appeared in apparently less quantity only until the minute 180th, which suggests that even though the PMNs from diabetic people formed NETs, as well as those from the healthy volunteers, the DEC delayed the NETosis (Figure 3). It is worth to note that quantitatively, the increase of the tendency toward NETosis in this group occurred 30 minutes before the healthy volunteer's group. Nowadays, there are no studies that define clearly the duration of the induction period toward NETosis when PMA is used as an *in vitro* trigger of PMNs from people with DM2. Possibly, this advance of 30 minutes is an effect of the concentration of PMA (600 nM) used in this study. Also, it is well known that diabetic patients have high levels of proinflammatory cytokines, which may act as NETosis inductors [27]. It is necessary to point that the chronic hyperglycemic state may play an important role in NETosis, which can be induced *in vitro* challenging PMNs with glucose at concentrations between 20 mM and 30 mM [28, 29]. This phenomenon could be happening in the hyperglycemic tissue microenvironment of diabetic people with bad metabolic control and might be potentiated by known NETosis inductors like LPS. However, there are also conflicting reports stating that under constant hyperglycemia, PMNs may become insensitive to LPS [30]. Other factors can contribute to the predisposition of PMNs of diabetic subjects for the formation of NETs, such as the overexpression of the enzyme peptidyl arginine deiminase 4 (PAD4) which is necessary for chromatin decondensation [17]. The possible interactions of the comorbidities in diabetic people could be studied because some of them, such as obesity, have been linked to NETosis in an isolated way [31].

## 5. Conclusions

In conclusion, the obtained evidence in the morphological and morphometric analyses of the present study suggests that both in healthy and in DM2 people, DEC exerts an immunomodulatory effect lowering and delaying the tendency toward NET formation by their PMNs. In the future, studies including longer observation times as well as higher doses of DEC will allow us to elucidate whether NETosis is only delayed or can be eliminated by the administration of DEC. Knowing that the presence of NETs retards the healing of ulcers on the feet of diabetics, this knowledge could open a therapeutic possibility for people who suffer diabetic foot ulcers.

## Data Availability

The data used to support the findings of this study are restricted by the COBICIS Comite de Bioetica en Investigacion en Ciencias de la Salud, CONBIOETICA 19-CEI-001-20170207, and COFEPRIS 103300538X0322. Protocol approved on May 11th 2017, registry number: 005-2017-01-JCSR, in order to protect [PATIENT PRIVACY]. Data are available from Dr. Juan Carlos Segoviano (drjuancarlos.segoviano@live.com.mx) for researchers who meet the criteria for access to confidential data.
